# Will the Russian war in Ukraine unleash larger epidemics of HIV, TB and associated conditions and diseases in Ukraine?

**DOI:** 10.1186/s12954-023-00855-1

**Published:** 2023-09-01

**Authors:** Samuel R. Friedman, Pavlo Smyrnov, Tetyana I. Vasylyeva

**Affiliations:** 1 NYU Langone Health, New York, NY USA; 2 Alliance for Public Health, Kyiv, Ukraine; 3 Division of Infectious Diseases and Global Public Health, UC San Diego, San Diego, CA USA

**Keywords:** Ukraine, War, HIV, TB, Big Events, Risk

## Abstract

The Russian war in Ukraine poses many risks for the spread of HIV, TB and associated conditions, including possible increases in the numbers of people who inject drugs or engage in sex work in the years ahead. Ukrainian civil society and volunteer efforts have been able to maintain and at times expand services for HIV Key Populations. The extent of mutual-aid and volunteer efforts as well as the continued strength and vitality of harm reduction organizations such as the Alliance for Public Health and the rest of civil society will be crucial resources for postwar efforts to assist Key Populations and prevent the spread of HIV, TB and other diseases. The postwar period will pose great economic and political difficulties for Ukrainians, including large populations of people physically and/or psychically damaged and in pain who might become people who inject drugs. Local and international support for public health and for harm reduction will be needed to prevent potentially large-scale increases in infectious disease and related mortality.

## Introduction

As McKee and Nagyova point out [1], after the war in Ukraine ends, months and years of neglect and scarcity of resources will create opportunities for new and existing serious health problems to flourish. Much of the efforts to prevent this will focus on care for the wounded and mentally traumatized, as well as efforts to prevent infectious disease outbreaks, consequences of extremely delayed diagnostics and care for chronic conditions, and environmental pollution from bombings, fires and the like. Unexploded shells and landmines on huge territory, including possible contamination with depleted uranium from shells used against tanks, will also threaten lives and health [2, 3].

Serious risk exists of a large-scale resurgence of the HIV/AIDS epidemic and of epidemiologically related epidemics during the war and in the postwar period. This paper will address these risks, and how to respond to them, using a Big Events framework [4–8]. This framework uses a transdisciplinary lens to understand how events like wars, revolutions, sociopolitical transitions, economic crises, natural disasters and pandemics can sometimes (but not always) lead to HIV outbreaks or outbreaks of related infections through re-shaping social and economic conditions, disrupting medical and governmental institutions and budgets, and producing personal experiences and traumas in ways that create social niches for the spread of sexually and blood-borne diseases and for an increase in overdoses among people who use drugs (PWUD).

A framework such as Big Events is useful in pointing to potential sources of vulnerability and strength for the health of the Ukrainian people. It thus can suggest both issues that need to be assessed now and over the coming years (perhaps including by sociobehavioural surveillance or by research) and also can help in devising appropriate programs and other responses to emerging issues. This framework informed our previous work on the transmission dynamics of HIV and Hepatitis C Virus (HCV) in Ukrainian people who inject drugs (PWID) displaced by the first wave of the Russian aggression in 2014, which showed that internally displaced PWID (IDPWID) got infected soon after displacement and that many of them are staying out of care [9, 10].

**Fig. 1:**
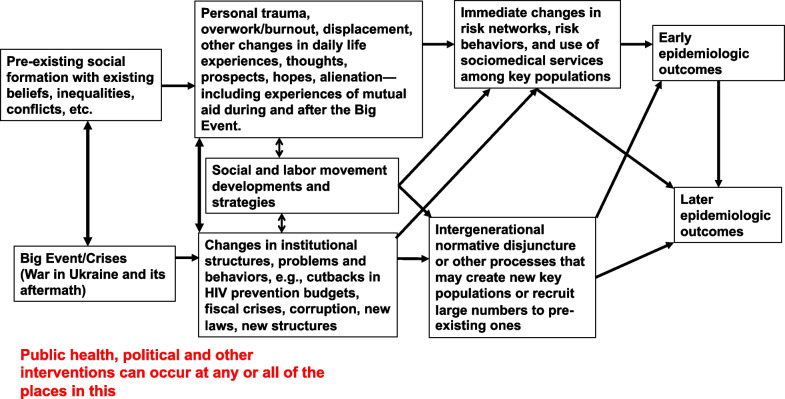
General model of Big Events processes end epidemiologic outcomes

Figure 1 presents a general diagram of this framework. The framework starts with the pre-existing social conditions in a country or other unit and the “Big Events” that take place there. These potentially affect the daily lives and problems of the population and the country’s institutional structures and policies, and these may lead to social movements or other struggles developing. These changes and struggles can then lead to short-term and long-term developments that shape HIV and other epidemics. In the short run, they can affect the risk and protective (e.g. health-seeking) behaviours and/or the social influence networks and risk networks of Key Populations such as PWUD, men who have sex with men (MSM), and sex workers—and depending on the extent of such changes, this may lead to an immediate increase in new infections or other health outcomes or create predispositions for future outbreaks. In the longer run, the changes in society may lead to a large-scale influx of people (and perhaps particularly of young people) into drug use, multi-partner high-risk sex or sex work. If this occurs, the size of the population at high risk of HIV infection can massively increase, and so can the HIV or other epidemics, similarly to how our previous analysis showed that HIV grew exponentially through large pools of new susceptible individuals in Russia and Ukraine in the 1990s [11].

## Background and setting

Importantly, when the war started, Ukraine already had a neoliberal economic and social system ruled to a large extent by politically connected “oligarchs;” cultural and linguistic differences based on a long history of being split among other empires and countries; large populations of PWUD and of sex workers; a sizable LGBTQ population that faced considerable stigma [12]; large HIV, hepatites and sexually transmitted infections (STI) epidemics among members of these populations [13–15]; and a sizable tuberculosis (TB) epidemic. TB may well flourish during and after the war [16]. Ukraine has the highest prevalence of multi-drug-resistant (MDR)-TB in Europe and third highest prevalence of extensively drug-resistant (XDR) TB globally [17]. *Beijing* strain MDR-TB, prevalent in Ukraine, is associated with higher virulence and increased transmissibility [18]. Since TB case-finding and treatment services are being interrupted in the territories that see military action and in the adjacent regions [19], and wartime destruction leads people to gather in poorly ventilated structures and to live in crowded conditions due to displacement and/or to housing destruction, the risks of a larger epidemic of MDR-TB, particularly Beijing MDR-TB, are high.

In the twenty-first century, unlike Russia, Ukraine also had developed an extended civil society-supported system of harm reduction and medical interventions that was holding the HIV epidemic in check [20, 21]. These programs were heavily financed by international donors, mainly The Global Fund to Fight AIDS, Tuberculosis, and Malaria (further—Global Fund) and PEPFAR. After the 2014 political revolution known as the “Revolution of Dignity”, this harm reduction system was seriously disrupted in the eastern parts of Ukraine and Crimea following Russian invasion. The war and occupation of Eastern Ukrainian regions and Crimea also led millions of people to leave the impacted areas and move to other parts of Ukraine, Russia or elsewhere, even before the escalation of the war in 2022.

## The Big Event

The full-scale Russian invasion in February, 2022, was followed by massive death and injury, considerable ecological disruption, massive attacks on power systems and the provision of fresh water, displacement of millions of people (mainly women and children) to outside of Ukraine (including the forced displacement of many to Russia) and of additional millions within Ukraine [22, 23]. It led to massive injury, pain and trauma to many civilians as well as combatants.

Displacement creates many social problems and also opportunities for the spread of HIV and other diseases. Evidence about the effects of displacement due to the war in Eastern Ukraine after 2014 describes what may be happening now on a much wider scale. A qualitative study conducted in 2020 showed that Ukrainian internally displaced persons (IDPs) reported challenges with social adaptation, isolation and relational conflicts within and outside families [24]. In the absence of a supportive environment, alcohol and other substances can be used as coping mechanisms [25]. This isolation is evident in data on HIV and HCV transmissions in Ukrainian IDPWID, which also showed that these transmissions mostly took place in networks of people who arrived from the same home regions [9, 10]. The support networks used by Ukrainian IDPs to obtain knowledge of new environments and resources, can become sources of drug procurement for PWUD.

As social support mechanisms are essential for recovery from mental health struggles, it is crucial for the ability to follow routines (including health routines) during postdisplacement adaptation [24]. Previous studies have indicated that health-seeking behaviours, including seeking HIV testing for those at risk for HIV and treatment for those living with HIV, receive lower priority than the fulfilment of basic needs, such as procurement of food, shelter and employment, in conflict-affected areas [26]. Importantly, potential reduction in antiretroviral therapy (ART) adherence can lead to the emergence of drug-resistance mutations (DRMs), which, if transmitted, can further result in a two–fivefold higher risk of virologic failure compared to infections with drug-susceptible viruses [27–30], further threatening HIV epidemic control.

## Changes in daily lives, institutions, policies and related social struggles

The invasion was met by the massive mobilization of Ukrainians who joined formal military forces, other forms of territorial defence or partisan units. It also led many millions of Ukrainians to engage in mutual-aid and support for those whose homes were destroyed, whose breadwinners were in the army, or who needed other forms of help. All of this disrupted some social networks, altered the content and intensity of others and created many new network ties among people who came to know each other. Some networks and organizations provide supplies and humanitarian aid for the army and those in need. These efforts are currently performed mainly by volunteers and are a potential training ground and organizing network for future projects to protect public health if pathways for long-term coordination and management are established. Other networks might create the basis for new or altered sexual or drug using networks and their accompanying risks for HIV and other infectious diseases, as well as their efforts to prevent such risks, although the extent to which such networks form is likely to be contingent on the course of the war, the social conflicts that may accompany or follow the war, economic conditions and much else [4, 7, 31, 32].

As in most if not all wars, the invasion created great strains at workplaces. Workers engaged in support for war-related efforts—including goods and services for the civilian population, particularly given the destruction wrought by the war—have undergone long hours of work. Although to some extent workers voluntarily spent extra time and effort to support their fellow Ukrainians, this nonetheless created great stress, exhaustion and in many cases burnout. Wartime laws and regulations limited citizens’ rights and gave management and employers extra authority to enforce their will on workers [33–35], which sometimes exacerbated these stresses and perhaps attacked the dignity of the workers. (These difficulties can often lead to stress or conflict in family relationships or friendships.) Many Ukrainians chose to join the army or were mobilised, creating gaps in the workforce which were often either filled with new hires or by having existing workers work harder. When soldiers leave the military and seek their old jobs back—as is currently legally guaranteed—those who lose jobs as a result might become a source of additional political conflict, particularly if economic conditions then are severe.

As in many disasters [36–39], massive volunteer efforts have taken place in communities to help those in need. This adds to the overwork—particularly when it involves observing or being part of the traumatic experiences of harms caused by bombs or shelling, or providing social support for victims of torture in previously occupied areas. This adds both to stress and overwork. Importantly, most of the volunteer effort is conducted and led by people who mobilize themselves independently of governments or employers. It is thus a social mutual-aid response to meet each other’s needs and to build a mutually shared destiny. As such, the mutual-aid experience is an important resource and training for social solidarity efforts such as harm reduction after the war.

## HIV risk practices of key populations

Even though HIV prevention services have continued to be provided during the war, surveillance data on the HIV risk practices of Key Populations are quite limited due to the difficulties of collecting such data. Nonetheless, some information is available. According to a report by the Alliance for Public Health that focused on people living with HIV (PLWH), MSM, PWID and other members of Key Populations [33], in the months immediately following the full-scale invasion in 2022, PWID have had experienced difficulty in some regions gaining access to sterile needles due to disruption of pharmacy supplies and evacuation of community needle and syringe points to safer areas, and the drugs they obtain through street sources have deteriorated in quality and increased in price. Further, the increased vigilance of people in the street and of police and other street patrols creates difficulties in accessing drugs. These difficulties might be even more pronounced for IDPWID since they are operating in unknown environments; our research conducted in Georgia showed that 92% of IDPWID who participated in the survey reported that their behaviours changed to riskier since their displacement [40]. Although available data do not suffice to accurately measure changes in syringe sharing (despite our communication with social workers that PWID continue to “practice drug use in the usual way”), these conditions would tend to increase it. Communications with local NGOs report that in areas of active hostilities and in areas occupied by Russian troops, syringe sharing has increased due to lack of needles and syringes supplies by local community organizations or pharmacies.

Furthermore, higher use of alcohol, smoking and being malnourished are risk factors for latent TB infection (LTBI) reactivation [41]. As > 20% of Ukrainians have latent TB [42], war-related conditions that can increase substance use and reduce access to food provision in particularly affected areas, create predisposition for LTBI reactivation and subsequent increase in TB incidence.

In the beginning of the full-scale invasion in 2022, in some parts of Ukraine and particularly in areas with historically high prevalence of sex work such as Odesa, sex workers faced increased competition among themselves due to high numbers of newly arriving internally displaced persons who engaged in sex work, who were more willing to offer services for reduced price, including providing more condomless sex (through communications with local NGOs). Sex workers there have also faced higher risk of physical and sexual violence during the war.

At the same time, war can also reduce HIV risks by reducing frequency of sexual intercourse, as is common during military conflicts, natural disasters and epidemics, due to increased stress and anxiety [43]. Similarly, in Ukraine MSM report that the war has led to less sexual desire and many have not had sex since before the war, even though this report is based on a small subsample of eight MSM in a national qualitative survey [44]. Furthermore, none of the eight MSM respondents in the survey reported cases of sexual or physical violence.

Importantly, in research reported by the Alliance for Public Health, many participants report that they have stopped receiving HIV counselling services, free condoms, HIV testing and STI testing. This suggests that many members of Key Populations may have reduced their adherence to antiretroviral therapy schedules, which together with ART drug supply disruptions documented in 2022 [45, 46] can further increase DRMs prevalence and compromise effective treatment as prevention. At the same time, DRMs surveillance is limited in Ukraine due to high costs of genotyping; thus, research into the effect of war and displacement on ART adherence and associated HIV drug resistance is necessary. Furthermore, the extent of undetected and untreated HIV and STIs may have increased. Both Key Population members and service providers report that Hepatitis C services (diagnosis and treatment) have become very difficult to obtain.

## Responses by harm reduction organizations during the war

After the war escalated in 2022, most civil society and community organizations who were working in the field of HIV response immediately adapted their existing HIV prevention programs [44]. NGOs serving PWUD report that the demand for sterile syringes and condoms increased (due to interruptions in the supplies reaching pharmacies and due to worsened economic situations of clients, as well as to clients wanting to stock up in advance in case the war led to their unavailability). The local non-government community organizations, Center of Public Health of the Ministry of Health of Ukraine and the Alliance for Public Health, using supplies made available by the Global Fund, were able to provide increased prevention materials to meet Key Population needs. These agencies intensified their use of mobile vans to deliver services and switched to online counselling whenever it was possible. Organizations also started new components to address the needs of clients and other community members. Most NGOs started to provide humanitarian support (food, hygiene, clothes including for children of clients), established temporary shelters for clients who moved in from other regions or lost their homes and evacuated many clients and personnel from the active fighting and occupied areas. With intensified Russian shelling of cities and towns across all Ukraine, many projects requested bullet-proof vests and helmets for their workers to continue service provision as well as autonomous electrical power solutions, electricity generators and power banks. Mobile vans of the Alliance for Public Health were rapidly mobilised to deliver medicine (including government-procured methadone), harm reduction materials (syringes, needles), HIV rapid test kits, general medical equipment and humanitarian aid to areas around the country not accessible by any other logistic and transportation means.

## Changes in size of key populations

Major social crises often lead to increases in sex work and drug use and their associated diseases [4, 7, 47–49]. After the break-up of the Soviet Union in the early 1990s, many of its former components—and perhaps particularly Ukraine—saw massive increases in drug use, alcoholism and sex work. These led to gigantic epidemics of HIV, TB and STIs, and to many deaths. Similar increases in HIV took place in South Africa after the end of apartheid and in Indonesia after the overthrow of the Suharto regime. Such increases due to what have been described as “Big Events” are contingent (that is, it does not always happen).

Local NGO staff and healthcare providers in Ukraine report that the war is increasing the risk factors for HIV and related diseases, mostly due to people being displaced, suffering from lack of proper support, employment, destruction of healthcare facilities and evacuation of healthcare and community workers from some areas. Many displaced people—particularly women—are engaging in sex work as their best available or only income source. The number of potential clients has increased because many women and children left the country, but this was forbidden for men, and military struggle means that soldiers and to some extent militia groups are away from their regular partners.

Emerging data (available through communications with local NGOs) suggest that in Odesa, and perhaps elsewhere, some displaced people are beginning to use drugs, perhaps in part due to the stress of war as well as displacement. Pain from war-related injuries or psychological trauma may lead some soldiers, ex-soldiers and civilians to begin to use drugs, perhaps as the aftermath of opioid pain medications following injury. With time, homelessness might increase in IDPs as family or friends become less willing to house them. Our previous work with IDPWID in Odesa showed that unstable housing (including homelessness) was associated with more recent injection drug use and that its prevalence grew with time among IDPWID [9].

## Implications of Big Events framework for what may happen after the war ends

Postwar developments will occur in the context of these pre-war and wartime experiences, social structures and epidemic realities. Much will depend on the war’s geopolitical outcome and on the extent of damage and trauma that Ukrainians undergo. In addition, whether the war is followed by financial efforts to enforce the debts Ukraine has incurred, or, conversely, by forgiveness of debts and additional monetary and material aid, will shape many but not all of the pathways indicated in Fig. 1.

There is thus a degree of uncertainty in what we say below. Part of this uncertainty comes from not knowing how, when or if the war will end, and part comes from not being able to predict how populations will react to the political, social and economic difficulties of the postwar period. What we write below is most germane to areas that end up within the borders of a relatively stable postwar Ukraine. Areas that end up under Russian rule may face additional problems depending on Russian policies in conquered territories, the degree to which insurgency takes place, and related reprisals. A crucial factor for the future of the areas within Ukrainian control will be the extent to which debt forgiveness occurs and the extent to which lenders demand that austerity and structural adjustment programs be implemented—all of which will tend to increase the likelihood and magnitude of drug use, sex work and the epidemics discussed below.

For example, should the war be followed by widespread unemployment, this may lead to widespread demoralization, scapegoating of members of Key Populations and perhaps to two forms of normative disjuncture: 1. between adults and teenagers who become a “lost generation” unable to see a future for themselves; 2. between those with disabling physical or psychological wounds or trauma and those who are more able to function in postwar society. In either case of normative disjuncture, those excluded may engage in high-risk drug use or sex.

Based on past history, the end of the war will see considerable demobilization of the military and the return of many of its members to civilian employment. This may lead to considerable social struggle. If these struggles are major and prolonged, this may lead politicians, the state as a whole and/or employers to engage in divide-and-rule politics in which they seek scapegoats on whom to blame the ills of the society and economy [50]. Convenient targets for such scapegoating may well include Key Populations like PWUD, gay and bisexual men, transgender people and sex workers—especially in Ukraine where stigmatization of these populations is still relatively high [51, 52]—which would make programs to prevent and treat HIV and related diseases harder to conduct and to get funded.

Such efforts at scapegoating may be lessened, and efforts to prevent and treat HIV and related diseases strengthened, to the extent that the wartime experience has strengthened feelings of social solidarity among much of the population, and to the extent that such scapegoating can be labelled as akin to the Russian way of doing things, and increased tolerance towards marginalized populations can be framed as a move towards European values of human dignity and human rights.

These dynamics will affect the nature of people’s daily life experiences, thoughts, prospects, hopes and alienations. They will also affect the extent to which the traumatized deal with their trauma through drug or alcohol use or in more socially cohesive ways. One important possibility is that some otherwise traumatized people will find social integration and support through participation in social activities or movements. (This could include getting involved with harm reduction efforts, with building or supporting LGBTQ community projects, with labor struggles, or, contrariwise, with scapegoating and reactionary politics.)

These issues will shape the extent to which medical and social services, including harm reduction and HIV and drug treatment, decrease, are restored or are increased. They also will shape the extent to which war-time sex workers can find alternative employment.

If the war is followed by widespread and sustained economic hardship, cutbacks in social programs and support of civil society—which could result if foreign debt is not forgiven, for example—this could lead to a situation like that which prevailed in Ukraine during the 1990s in which drug use, alcoholism and sex work become widespread. (To some extent, this might be particularly likely to take place among people who were teenagers or street children during the war. As we have discussed in previous papers about Big Events [7, 53, 54], youth and street children are particularly likely to undergo normative disjuncture from dominant norms during and after Big Events.) The extent of physical and psychological trauma in the population makes this particularly likely. If this occurs, maintaining and strengthening a wide variety of harm reduction efforts of HIV treatment as prevention, and of expensive treatments for MDR-TB and XDR-TB [55], will be critical.

Pre-war Ukraine had harm-reduction and policy-focused organizations of PWUD, of LGBTQ people, of sex workers and of PLWH. During the war, these organizations have continued their efforts and tried to scale-up their activities to cover more geographical regions and include IDPs. These efforts are complicated by the fact that many members of these organizations have themselves been displaced, continuing their activities from other parts of Ukraine or abroad. Little is known, also, about the extent to which members of these Key Populations have taken part in the war effort and/or the mutual-aid efforts of civilians, although some data suggest that some members of Key Populations have been active as volunteers [44]. To the extent to which they have held together and been part of mutual aid, they will be in a stronger position to help with disease prevention, care and advocacy in the postwar period.

## Postwar changes in risk networks, risk behaviours and use of sociomedical services among Key Populations

One important aspect of this may be the extent to which newcomers to these populations join pre-existing risk networks or maintain separate risk networks and engage in similar risky and protective behaviours. Taking part in networks with older PWUD, sex workers or MSM can be valuable in terms of intergenerational transfer of knowledge about safer behaviours, skilled assistance in coping with overdoses or other urgent threats and gaining access to needed services. On the other hand, since the longer-term members of these populations are more likely to be infected with HIV, HCV or other long-lasting infections, being part of their networks may increase new initiates’ risk of becoming infected. Our previous phylogenetic analysis, based on a small number of HIV sequences obtained from IDPWID in one region of Ukraine, showed limited mixing of HIV viral lineages between local and displaced PWID communities [9].

The war has disrupted supply of drugs and of harm reduction materials in some geographical areas. Postwar shortages in harm reduction materials could occur due to economic or political problems or if donor support is reduced. Changes in drug supply in postwar period are hard to predict. All of these can affect the extent of injection versus non-injection use, of sharing injection equipment and of service usage.

The war has also disrupted HIV, HCV, TB, STI and other forms of case-finding, diagnosis and treatment in many regions, mostly due to reallocation of community and healthcare workers and organizations from some places (data available through communication with healthcare workers). There is a strong likelihood that despite all the efforts many PLWH who had kept HIV viral load suppressed no longer do so, and that large numbers of cases of a number of infectious diseases remain undiagnosed and untreated.

Both the war and the socioeconomic changes in the postwar period may disrupt the social cohesion of drug scenes and networks. The return of many women and children who fled from the war, and the emigration of men once controls are lifted (perhaps to join family members who fled), may shake up risk networks and venues.

Wartime and postwar politics could increase the social isolation of Key Population members from others. If it leads to more drug use in solitary circumstances, this could lead to an increase in fatal overdoses.

On the other hand, to the extent that the wartime struggles and mutual aid, or postwar social tendencies, increases the integration of Key Populations such as PWUD, men who have sex with men or sex workers with the rest of the population, this could lead to reduced risks and less stigmatization [56]. We would anticipate that favourable trends will be strengthened to the extent that Key Population members take part in military and mutual aid efforts during and after the war.

The reverse, however, is also possible. There may be increased social distance from non-users as shirkers or due to stigma. This could lead both to increasing attacks on their human rights and greater difficulty in getting them diagnosed and treated early in their course of infection.

Some PWUD report that they quit using drugs as the result of wartime needs or experiences. Some may have quit during the war, but begin using drugs again after it ends. The proportion who begin again will be shaped by the extent to which harm reduction services are maintained as well as by how Ukrainian society and politics develop after the war ends.

And as discussed above, there is a real danger of large-scale increases in the numbers of PWUD, PWID and people who engage in sex work—which is also contingent upon what happens during and after the war.

## Thoughts about solutions

When the war ends, even under the most optimistic scenarios, Ukraine will face a massive rebuilding task. This will be very expensive, and obtaining the needed resources could create enormous debt burdens and requirements to engage in “structural adjustments” that impoverish many people and that cripple public health and social service provision, at least in a short-term perspective. Public resistance to such structural adjustments is likely to take place and may succeed in helping to protect health, education and social welfare services that might otherwise be reduced or eliminated.

During the war, there has been a powerful self-mobilization of Ukrainians to fight Russia and to provide volunteer assistance and mutual aid to each other. The solidarity and skills built in this process form both a resource in resisting belt-tightening and a resource that can address a number of public health tasks when (and before) the war ends. In this concluding section, we will focus on these public health tasks.

Many public health campaigns before the war focused on individuals and their behaviours. It should be recognized that many of the health problems that Ukraine will face after the war ends will not be solvable with such individual-focused programs, useful as they are. Consider TB, for example. Individually focused efforts to restore easy access to screening for TB will be useful, and if a large TB epidemic takes place will need to be expanded to widespread active screening, perhaps based around the use of mobile vans (as is already being done in localities that have recently been liberated from Russian occupation). To bring a large TB epidemic under control, however, may well require large amounts of well-ventilated housing, but it is not currently available and its rapid construction at affordable prices seems unlikely. This would be an extremely expensive undertaking, further complicated by the likely increase in MDR TB, so it may depend on the outcome of popular organizing and activism to prevent structural adjustments, to acquire massive foreign subsidies or to re-purpose existing infrastructure.

The war has created a variety of forms of physical and psychological trauma and pain in large numbers of people. Many of these people may start using psychoactive drugs and/or alcohol to deal with the symptoms of trauma. This, in turn, may put them at risk of HIV and other sexually or blood-borne infections. It should be feasible to build on forms of mutual-aid voluntary self-organization created in neighbourhoods and workplaces during the war to establish local social support for the traumatized and to encourage pain alleviation practices that are less likely to lead to HIV or other infections. Such social support can mitigate risks of substance use disorders and mental health problems by directly affecting people’s resilience to stressors [57]. In some localities and situations, this may be the only form of available support for people who might have also benefited from referrals to professional care if such care were available.

Organizations such as the Ukrainian Alliance for Public Health and “100% Life” developed ways to use mobile vans like HIV outreach and diagnostic stations before the war. During the war, they expanded their use to include other forms of health provision. It may be useful to further expand these efforts. This might include a large fleet of mobile outpatient clinics to provide basic health and dental services (+vaccination), gynaecological screening, mobile infections diagnostics (including HIV, TB, hepatitis, sexually transmitted infections), assessment, short interventions and referrals for substance use disorders and mental health problems, provision of outpatient treatment and social and psychological support along with humanitarian and hygiene aid.

Outreach efforts of all sorts, including those using mobile outreach, should work with locally based mutual-aid and other groups to support Key Population members (including those who may have engaged in drug use or sex work while in other countries or while displaced within Ukraine) and those at risk of engaging in sex work or beginning to use drugs. This may be particularly valuable for assisting people with trauma, whether psychological or physical.

Vaccination and diagnostic catch-up campaigns will be needed. Many people in Ukraine have missed recommended screenings and vaccines during the war. Mutual-aid groups, mobile vans, and other efforts should be assisted to engage in vaccination and screening efforts.

As we have emphasized previously [7], sociomedical surveillance systems will be needed both to detect emerging infectious disease outbreaks and to foresee and understand the emergence of high-risk networks and behaviours. Ukraine saw the rise of such networks and behaviours in the 1990s when injection drug use and sex work increased and became a major focus of disease transmission. Mobile clinics and other diagnostic centres should be used to detect infectious disease outbreaks as early as possible. In addition, it will be important to conduct social surveys (and appropriate qualitative social field research) among potential high-risk populations including youth and people traumatized by the war who may be particularly likely to use drugs. In addition to standard psychosocial screening tools, we recommend the use of measures of the kinds developed for Big Events in the NIDA-funded “Measures Project” [53, 54, 58–61].

## Conclusions

The people and organizations of Ukrainian civil society have done a remarkable job supporting each other during this war. Although less salient than their efforts to support the military and those injured, displaced or otherwise made homeless due to the war, their efforts to provide needed services and supplies and to support human rights of PWID, PLWH, sex workers and men who have sex with men have been widespread and remarkably effective. Research is needed, however, to determine the extent to which these efforts have succeeded in preventing high-risk behaviours and related infections among these populations.

When the war ends, it will be important to maintain the strength of volunteer efforts, mutual aid, non-governmental organization and other civil society programs to assist these Key Populations. Support will also be needed for the many thousands who will suffer from physical and psychological pain and trauma for many years to come. Ongoing surveillance of the extent to which they turn to street medications including pain relievers and other psychoactive drugs will be needed, as well as harm reduction and treatment programs for those who begin to use drugs.

Ukraine is almost certain to undergo a considerable period of economic hardship, and perhaps of service cutbacks, and of social struggle around these. These will make it harder to prevent transmission of and disease and death from HIV, Hepatitis C, TB and other related diseases. Hardship may lead to an increase in the number of people who trade sex for money or other goods, with its concomitant disease risks. If large numbers of people, and perhaps of youth in particular, become cut off from the rest of society or from hopeful economic futures, this may lead to large increases in group sex scenes and drug parties. In such circumstances, some groups in society may urge increased policing and punishment as a solution. This is the path that Russia took in the 1990s and since, resulting in particularly high rates of injection and non-injection drug use and HIV transmission [4, 11]. In the USA, drug arrests have not been associated with lower rates of injection drug use and have been associated with higher rates of HIV infection, AIDS and AIDS mortality among PWID [62–64].

Harm reduction programs, combined with civil society support for the rights and health of often-stigmatized Key Populations for HIV and easy access to medical care, will be the only effective way to prevent the spread of HIV and other diseases in postwar Ukraine. Many international organizations, such as the Global Fund and PEPFAR, recognize this. Public health in the years after the current war ends will require both international funding and international and local social and political support for continued harm reduction efforts.

## Data Availability

Not applicable.
